# A Bibliometric Analysis of the Evolution of State-of-the-Art Blockchain Technology (BCT) in the Agrifood Sector from 2014 to 2022

**DOI:** 10.3390/s23146278

**Published:** 2023-07-10

**Authors:** Urvashi Sugandh, Swati Nigam, Sanjay Misra, Manju Khari

**Affiliations:** 1 Department of Computer Science, Faculty of Mathematics and Computing, Banasthali Vidyapith, Banasthali 304022, India; urvashi5sugandh@hotmail.com (U.S.); swatinigam.au@gmail.com (S.N.); 2 Department of Applied Data Science, Institute for Energy Technology, 1777 Halden, Norway; 3 School of Computer and System Sciences, Jawaharlal Nehru University, Delhi 110067, India; manjukhari@yahoo.co.in

**Keywords:** bibliometric analysis, blockchain, agriculture, food safety and security, agriculture supply chains, lens.org, VOSviewer

## Abstract

In recent years, BCT has garnered significant attention from researchers worldwide. The technology in question is a distributed database system characterised by its decentralised nature and lack of reliability. BCT has been widely adopted by numerous governments and scholars across various sectors for a number of years. Blockchain technology also involves highly innovative and advanced concepts. Given the increasing interest among scholars in the academic community regarding the agrifood supply chain, the objective of this study was to investigate BCT and its potential for application in the fields of food and agriculture. This research paper presents a bibliometric analysis of articles on the utilisation of BCT in the fields of food and agriculture. This study discusses scholarly articles that have been published in esteemed academic journals and conferences. Through our bibliometric analysis, we aimed to discern the recurring trends and themes within the research on BCT in relation to agrifood systems. Furthermore, this study examines a diverse array of research domains, prominent scholarly publications, leading publishing platforms, prominent funding institutions, and the prospective trajectory of future research. This study also presents the prominent patterns and themes within this field through an analysis of the most influential scholarly articles, authors, countries, and keywords found in the existing literature. Hence, this research employed various analytical techniques, including analyzing the co-occurrence of author keywords, bibliographic coupling analysis, network view map analysis, and co-citation analysis. This study holds promise as a valuable learning resource for aspiring researchers seeking to acquire compelling and pertinent information about research outcomes from studies on the utilisation of BCT in the field of smart agriculture.

## 1. Introduction

With each and every day, managing food supply for the world’s ever-increasing population becomes more and more difficult. Climate change, pests, floods, desertification, droughts, loss of biodiversity, and disease are the primary challenges regarding agricultural food managment. Some of these issues can only be addressed by making improvements to the agricultural processes we already use, which will make farming more appealing to smallholder farmers and hence more lucrative for them [1].

Many of the Sustainable Development Goals (SDGs) may be achieved with the use of information and communication technologies (ICTs). Some of agriculture’s greatest issues have been alleviated by the rapid rise of information and communication technology (ICT). The Internet of Things (IoT), increase access to mobile broadband devices, smart networks, drones, large data processing and analysis tools, and artificial intelligence are just a few of the recent developments that have given those in the agricultural field new tools and technologies to help them improve their production and marketing processes.

One of the most appealing use cases for blockchain is in agriculture, where it can help streamline the process of growing and distribuing food. Many current processes may be re-engineered using BCT, including facilitating transactions, identifying food origins, and establishing new interesting markets. In the agricultural value chain, BCT may minimize inefficiencies and save time and energy [2,3]. There is no denying that technology is essential in contemporary agriculture; however, increased efficiency could be achieved by exploiting the blockchain’s capacity to boost automation, digitalization, and food monitoring. Automated payments and self-executing smart contracts in agriculture are ready to revolutionize the industry. Crop insurance, green bonds, and traceability, in particular, will benefit greatly from the use of smart contracts [4,5]. BCT is becoming more popular in commercial agriculture and agribusiness. The expanded data management capabilities of emerging technology may help organizations streamline their supply chains and eliminate transactional friction [6].

This paper aims to provide a bibliometric analysis of the evolution of state-of-the-art BCT in the agrifood sector from 2014 to 2022. Bibliometric analysis is a quantitative method used to analyze the scholarly literature on a particular topic, including the publication output, citation impact, research themes, and collaboration patterns.

This paper seeks to answer the following research questions: What is the current state of research on the development of BCT in the agrifood sector? What are the primary research themes and topics related to the application of BCT in the industry? What are the use cases for BCT in agriculture? What are the challenges and limitations of the blockchain-based applications? What are the open research issues and the areas for future research? What are the field’s most influential publications, authors, journals, and institutions?

Overall, this study adds to the body of research on BCT’s use in the agricultural and food industries by conducting a thorough bibliometric analysis of the most recent studies on the issue. The study sheds light on the state of the field, the leading publications, authors, journals, and organizations, and the possible advantages and disadvantages of BCT in the business world. The study’s results may help direct future work on the subject and provide policymakers and industry stakeholders with insight into the potential benefits and drawbacks of incorporating BCT into the agrifood sector.

The present research article pertains to the domain of smart agriculture, wherein sensors are deemed to be instrumental in propelling advancements in this field. Precision agriculture, commonly referred to as smart agriculture, is a farming methodology that leverages cutting-edge technology and data-driven strategies to enhance agricultural operations and improve productivity. Sensors play a crucial role in smart agriculture systems by facilitating the acquisition of real-time data pertaining to crop growth, soil conditions, environmental factors, and livestock monitoring.

The remainder of this paper is structured as follows: In Section 2, we present a thorough assessment of BCT and its potential uses in the agrifood sector. The search parameters, data collecting procedure, and bibliometric indicators we used during our research are detailed in Section 3. The results from our bibliometric analysis, including output in terms of publications, citation impact, research topics, and cooperation patterns, are presented in Section 4. In Section 5, we discuss our research findings and how they relate to the aforementioned research questions. Suggestions for future studies, as well as the ramifications of the results of our research for the agricultural and food industries, are presented. Section 6 is the final section of this article, which houses our conclusions.

## 2. Literature Background

Finance, healthcare, supply chain management, and the energy sector are just a few of the areas that have been transformed by the rise of BCT. Researchers, decision-makers, and business leaders have all given this distributed ledger technology a lot of attention because of its potential to increase efficiency, security, and transparency across a range of industries. The use of BCT in the agrifood sector has grown in popularity in recent years as academics and industry professionals have come to realise its potential for solving significant problems frequently experienced in the field.

The agrifood supply chain must be transparent and traceable in order to guarantee the safety, quality, and authenticity of food products. Tracking the production, processing, and distribution of food items is challenging due to the old centralised systems’ frequent limits regarding the provision of real-time information. Additionally, the current procedures can be vulnerable to dishonest practises such as fraud or the use of false labels, which might jeopardise customer safety.

A decentralised, immutable ledger made possible by blockchain technology allows for the safe, open recording of transactions and data. BCT offers a tamper-resistant and auditable record of all operations throughout the supply chain by using cryptographic methods and consensus procedures. Any and all activity, including the transfer of commodities, changes in ownership, and quality control checks, is documented in a block, resulting in an unbreakable chain of data.

The potential advantages of using BCT in the agrifood industry have been noted in a number of studies. For instance, [7] performed a comprehensive analysis of BCT applications in the food supply chain and identified better traceability, improved food safety, decreased fraud, and greater customer confidence as the main benefits of BCT. They emphasised how the BCT’s immutability promotes confidence among stakeholders and allows for effective recall management in the event of foodborne disease outbreaks or product recalls.

Furthermore, Ref. [8] examined the use of BCT in agricultural commerce and discovered that it may expedite procedures, eliminate middlemen, and promote efficient and secure transactions. This research study also noted the potential of BCT’s smart contracts, which automate payment settlements and guarantee honest business dealings between buyers and sellers.

Although the potential advantages of BCT in the agrifood industry are encouraging, it is crucial to have a thorough awareness of the current research environment. A biblimetic analysis may assist in identifying the unresolved questions, themes, and problems associated with BCT’s use in agriculture. This study intends to add to the existing body of research in the literature by offering insights into the development of BCT research in the agrifood industry from 2014 to 2022 by presenting a bibliometric analysis.

Crop management, supply chain traceability, quality assurance, and sustainability are a few of the well-known study areas that were considered in our analysis. In this study, the worldwide cooperation and information diffusion in this field is highlighted through the identification of significant academic publications, authors, and nations. The study also presents the publications that are mentioned the most and highlights the significant contributions that have influenced the growth of BCT applications in agriculture and future research directions.

This study will inform readers about the present status of BCT in the agrifood industry by synthesising the available literature and identifying research trends. The results will be helpful for academics, decision-makers, and business professionals who want to use BCT to solve difficult problems in the agrifood sector.

## 3. Materials and Methods

**Figure 1:**
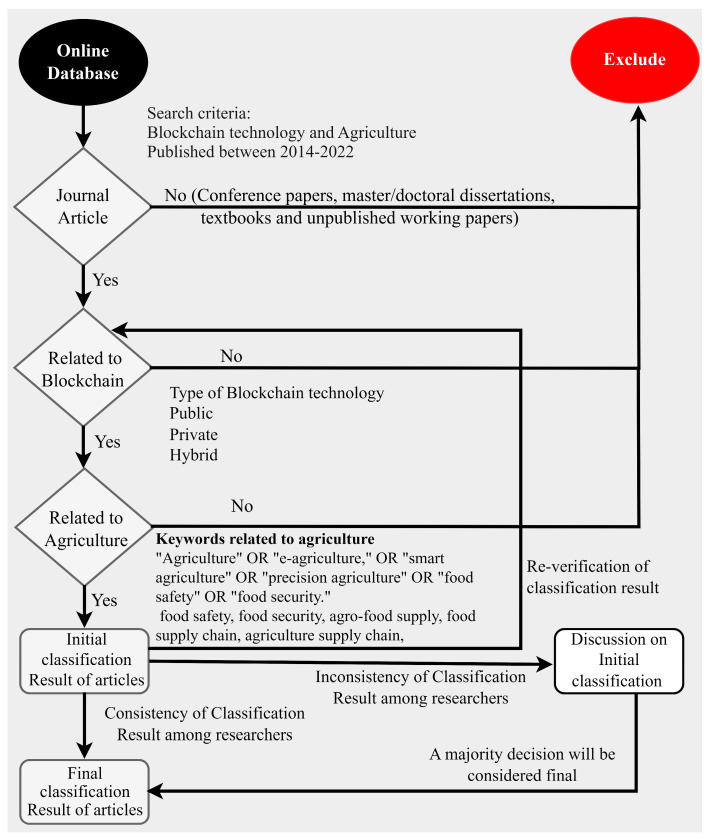
Research framework.

Firstly, a search was conducted across bibliographic databases that use search terms or keywords. Throughout the course of our investigation, we consulted a number of different scientific databases. These included ACM, IEEE Xplore, PubMed, the Web of Science, and Scopus. Figure 1 shows the research framework for our research methodology. We believed that, by picking these databases, we could hone in on reliable articles that had been published in reputable journals and conferences as opposed to gleaning generic information. We searched for information in the databases using the phrases “blockchain” AND “Agriculture” OR “e-agriculture,” OR “smart agriculture” OR “precision agriculture” OR “food safety” OR “food security”. The aforementioned search string was selected after conducting a pilot search in which we tested some commonly used terms and acronyms related to agriculture, such as food safety, food security, agrifood supply, food supply chain, agriculture supply chain, and so on. The results of that search led to the selection of the search string used for the creation of this article. When combined with “blockchain”, none of these agriculture-related terms, derivatives, or acronyms produced any new results that were not previously returned by our search string. Consequently, we discovered that all of these were included in our search string. We also found that conducting a search for articles with the keywords “blockchain” and “agriculture” alone did not yield the desired results for some publications. These publications may not have included “agriculture”-related keywords in their metadata; however, they may have included other agriculture-related phrases such as “weather crisis”, “environment”, or “transparency” in their metadata. When “blockchain” was searched for, “other options”, such as “distributed ledger technology”, did not provide any further results.

Due to the fact that this research topic is still relatively novel, the literature search was carried out without taking time constraints into consideration. As a consequence of this, every piece of information that was found in this field was considered relevant to our investigation, irrespective of the amount of time it took to find.

### 3.1. Screening of Relevant Papers

The articles that had been retrieved from the databases in line with our search strategy were subsequently subjected to a filtering process to determine whether or not they were pertinent to the topic at hand. The very first stage of this processing procedure consisted of examining the titles of the papers to determine whether or not they were relevant to our analysis. Any articles that had been retrieved but whose titles made it abundantly clear that they were not pertinent to our study were dismissed. Some of the articles that our search protocol found did not have anything to do with the use of BCT in the agricultural industry; hence, we did not consider such articles to be relevant to our analysis and removed them from consideration. In cases where the relevance of an article could not be determined based on its title, the article was moved on to the subsequent phase for further screening. The second stage of the screening procedure consisted of reviewing the abstracts of the papers that had successfully passed the first stage of the screening process. When determining whether or not a paper met our criteria for exclusion, it was sometimes necessary to read not only the beginning but also the end of the paper.

The following publications were not considered: (1) papers that have not been subjected to a peer review process, such as interview sessions and media news releases; (2) articles wherein the complete text has not been made wholly accessibile; (3) documents not mainly focusing on the use of BCT in agribusiness; (4) redundant documents; (5) articles not published in English; and (6) articles that have been withdrawn from publication. Papers that did not meet these exclusion criteria were considered mostly focused on the application of BCT in agriculture and moved on to the subsequent stage of the screening process.

### 3.2. Keywording from Abstract

This step organized relevant material into logical groupings. The process involves collecting keywords and concepts from article abstracts that reflect the publications’ area contributions. Keywords grouped the papers. Each document was checked to determine whether it belonged to a different category after categorizing them. The categories were changed if the document was deemed more suitable for a different category. When a paper did not match any existing category, a new one was created. Consequently, related articles were mapped into many categories.

## 4. Bibliometric Analysis

This section presents the results of our bibliometric analysis. Our search strategy extracted 465 scientific database documents. After the title screening, 192 items were eliminated. A total of 273 articles required further screening. Our search protocol uncovered publications that mentioned agriculture as a non-financial use case of blockchain technology. Combining the 273 Mendeley articles removed duplicates, reducing the selected papers to 173. We subsequently reviewed the abstracts, introductions, and conclusions of the selected articles to evaluate whether they met the aforementioned criteria for further screening. Hence, 130 articles were considered. Twelve additional non-agricultural articles were deleted after reading all of the selected items. These papers only partially mentioned the potential application of BCT in agriculture and did not provide any new ideas or concepts. After filtering, we were left with a total of 118 articles.

### 4.1. Bibliometric Analysis and Article Distribution

**Figure 2:**
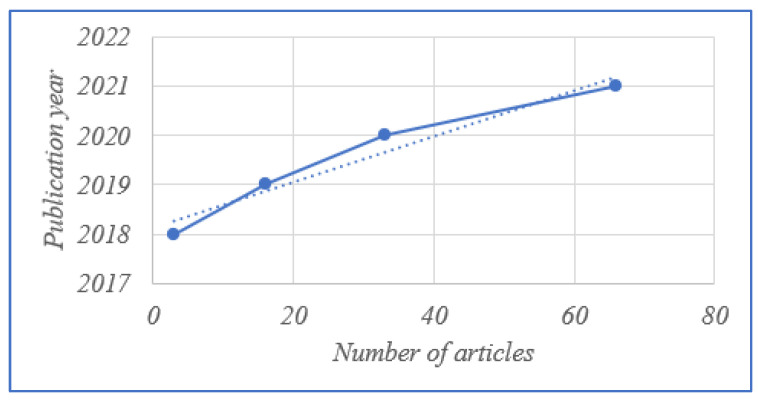
Publication years of the papers.

Although our search methodology did not include a time constraint, all of the articles selected were published after the year 2018. As shown in Figure 2, the distribution of the chosen articles is dominated by papers published in 2021, i.e., the majority (66 percent) of papers were published in 2021. Overall, 33 of the 118 articles chosen were published in 2020, and only 16 were published in 2019. Because the number of publications has increased steadily since 2018, it is clear that research into the application of blockchain technology in agriculture is topical and expanding fast. In fact, the number of publications has increased consistently since 2018.

**Figure 3:**
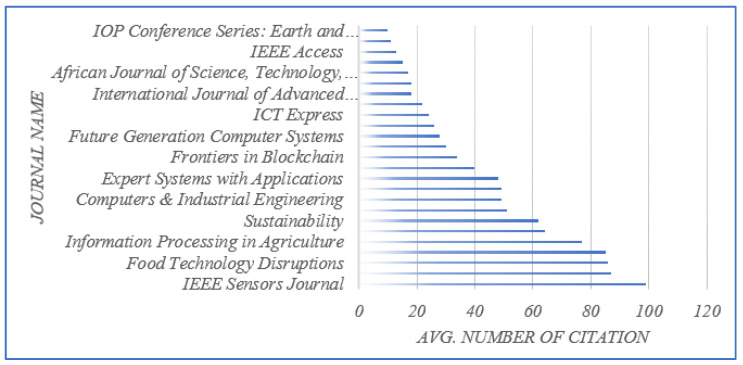
Top 13 journals based on the average number of citations.

**Figure 4:**
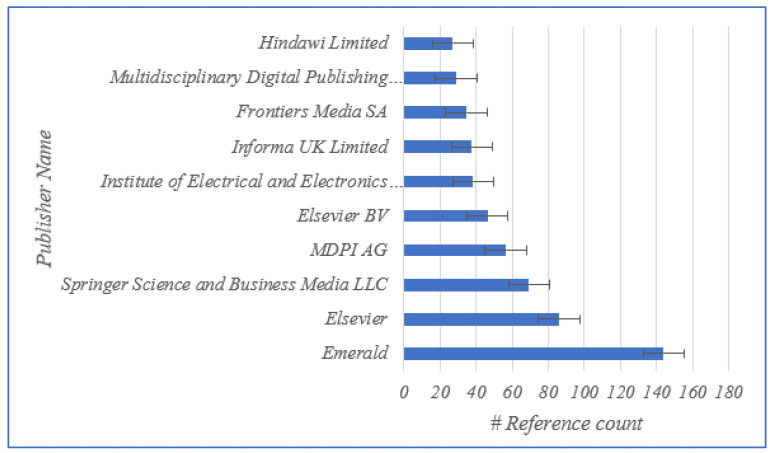
Top 10 publishers based on publication reference count.

**Figure 5:**
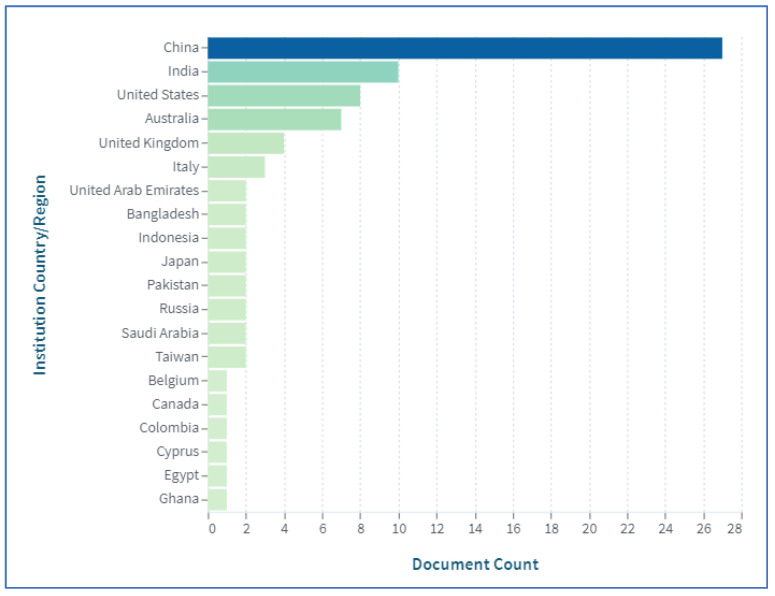
Distribution of papers according to the countries of the authors.

In addition, the journals of the articles that were evaluated are shown in Figure 3. To determine whether the authors of a given paper were affiliated with academia, industry, or both at the time of publication, the journals of the publications was a key indicator. The top 10 publishers are shown in Figure 4. In order to gain a sense of the geographical distribution of the members of the research community who were contributing to the research on the application of BCT in agriculture, we noted the locations (countries) of the institutions with which the authors of the selected papers were affiliated. We used the country of the corresponding author in cases when the authors of a particular work were from different countries. If the country of the corresponding author could not be determined at the time of publication, we used the country of the first author instead. You can see the geographical spread of the research contributors is shown in Figure 5. In our bibliometric analysis, we also examined the citations used in papers to identify the degree of connection between pairs of nodes (articles) in the 114-node network. This indicated the degree of connectedness between pairs of nodes (articles).

**Figure 6:**
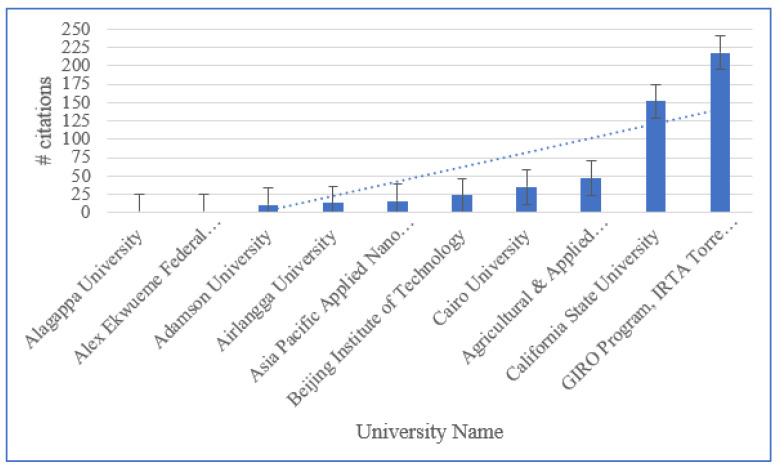
Top 10 institutions based on the number of citations.

**Figure 7:**
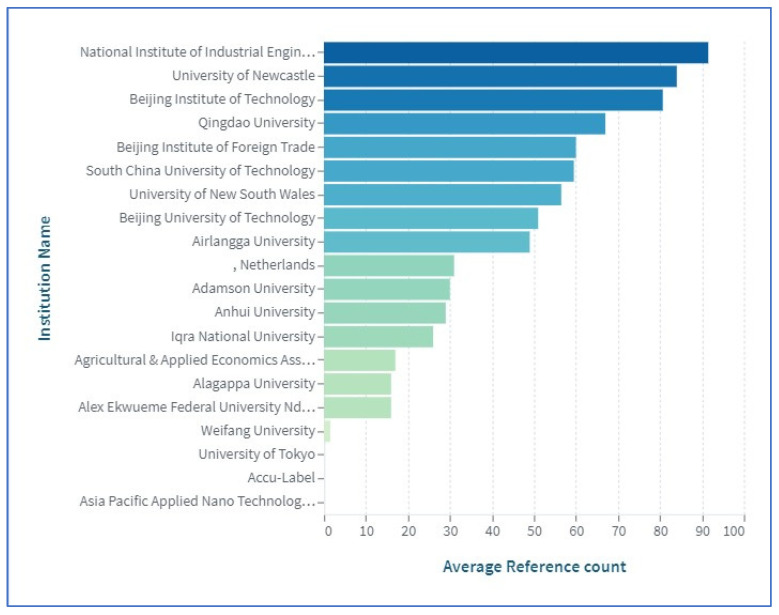
Top 20 institutions based on reference count.

Figure 6 shows the number of publications reported by various institutions and the quality of different institutions’ publications (assessed by measuring their number of citations). Additionally, Figure 6 also indicates which of these institutes is now in operation. Figure 6 and Figure 7 list the institutes that have undertaken research on the use of BCT in agriculture. Figure 6 demonstrates that GIRO Program, IRTA Torre Marimon, Barcelona, Spain, has the greatest number of citations. Based on Figure 7, the National Institute of Industrial Engineering is the institute with the greatest number of reference counts. Figure 6 and Figure 7 show that institutes in China have more citations and greater reference counts. China is home to some of the most prestigious academic institutions in the world. It seems that the place in which information on the use of blockchain in agriculture is being published far quicker than anywhere else in the world. Consequently, there seems to be fierce rivalry among universities across China in terms of publishing.

**Figure 8:**
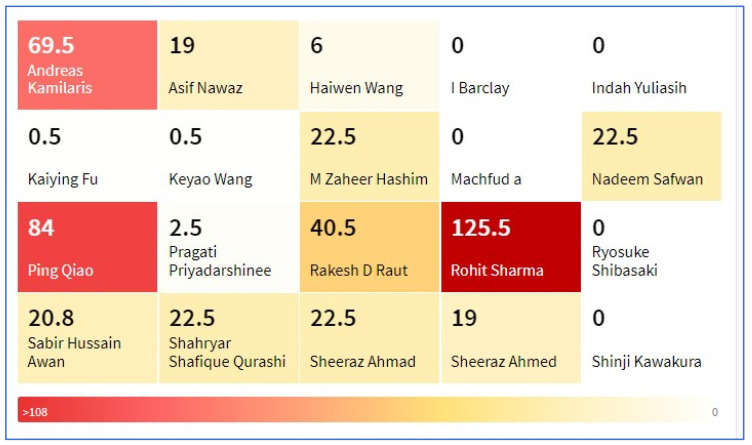
Heatmap of the most active authors according to avg. reference count.

**Figure 9:**
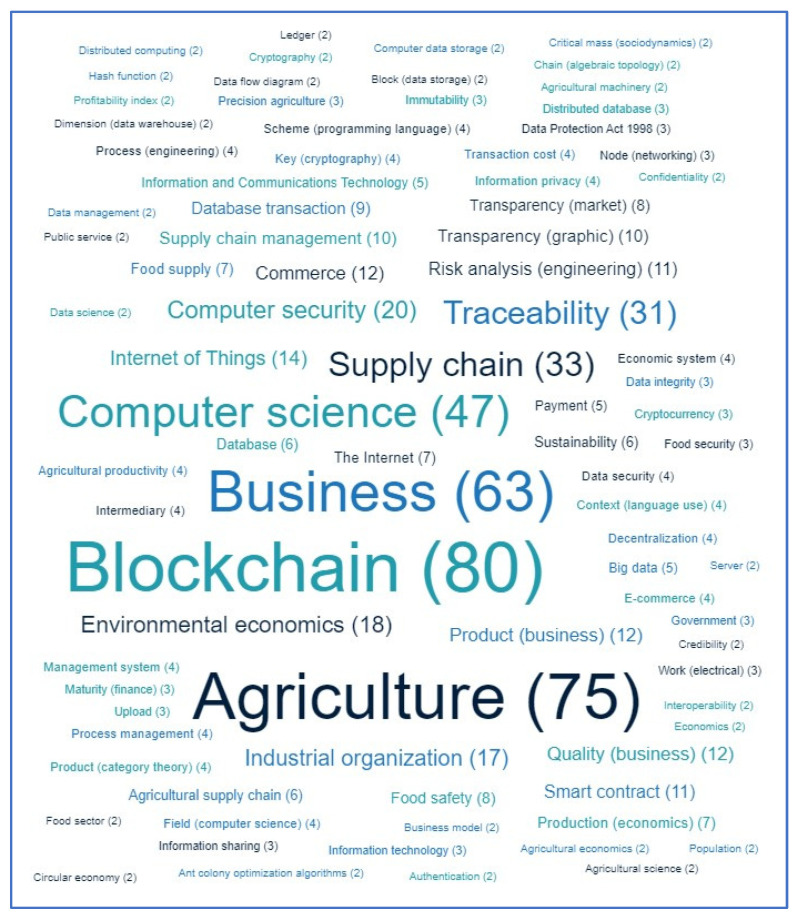
Word cloud of author keywords.

Figure 8 shows a heatmap of active authors according to the average reference count of their articles. It can be seen from Figure 8 that Rohit Sharma has the highest reference count. Figure 9 shows a word cloud of all of the keywords used by different authors in their articles. In total, 101 unique keywords were used. The most used keyword was “blockchain”, which was used 80 times, followed by “agriculture”, which was used 75 times. Table 1 shows the top 15 institutes by country. Table 2 shows the top 10 funding agencies in the studied research area.

**Figure 10:**
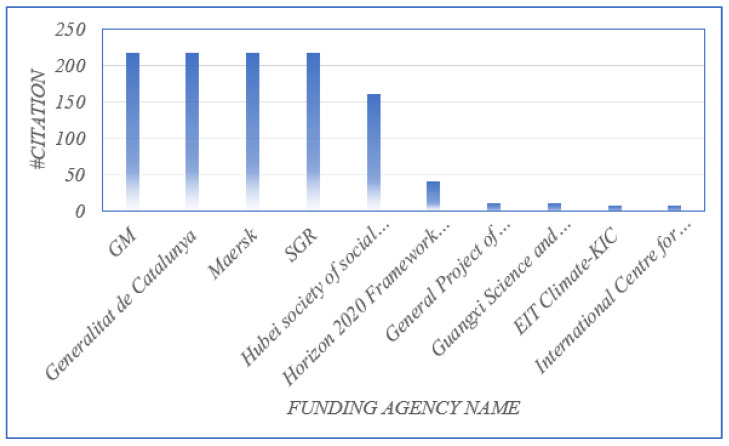
Top 10 funding agency based on publications citations.

Researchers who have restricted access to research funds, such as those working at less well-funded institutions, those living in developing countries, and less-experienced academics, are encouraged to begin new studies with the help of a research fund. There are several agencies and organizations all over the world that provide financial support for research. Figure 10 shows the top ten funding agencies based on the number of times they were cited as a source of research funds. Our analysis found that works supported by GM funding had the most citations. Table 3 shows the top fields of study in terms of document count.

**Figure 11:**
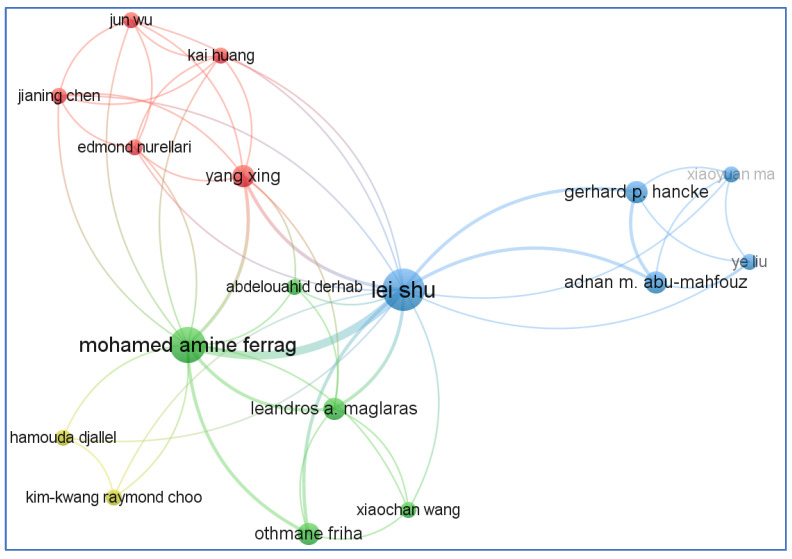
Co-authorship network (created with VOSviewer).

It is possible to describe scientific cooperation as the connection that occurs within a social context among two or more scientists to allow for the exchange of ideas and the completing tasks to achieve a mutually agreed-upon aim. Examining co-authorship in scientific and technology partnerships can elucidate the patterns of collaboration between people and organizations. A formal declaration of the participation of two or more authors or organizations in a technical document is made when a technical document is co-authored. Despite the ongoing controversy about the meaning and interpretation of co-authorship analysis, it is still commonly employed to investigate and evaluate patterns of scientific cooperation in the field of a given research topic. Co-authorship networks are nodes that represent authors, organizations, or nations that are linked together because they are co-authors responsible for the creation of a single publication. Figure 11 depicts a co-authorship network with respect to the existing literature on the use of BCT in the agrifood sector.

**Figure 12:**
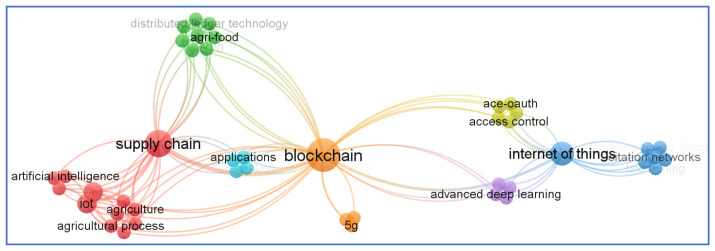
Co-occurrence of author keywords (created with VOSviewer).

Figure 12 depicts a clustering of the most commonly used keywords based on their co-occurrence. Colored clusters are formed by keywords that are frequently used together, while geographical distance reflects the relationship between keywords and lines represent the co-occurrence of keywords. As a cut-off value, a minimum number of one occurrence throughout publications (*n* = 19) is selected as a starting point. The size of a node is determined by the number of times a keyword appears. With co-occurrences represented by lines connecting nodes, the distance between nodes shows how closely the terms are connected to one another.

Using direct citation and co-citation analyses, citation analysis may be displayed as a network analysis to investigate the link between publications in a given time period. This can help to discover prolific publications and assist in analyzing the effect of an article. Various citation analysis software packages such as HistCite, Pajek, Gephi, and VOS viewer are available for download. Apart from Pajek, which is limited to “Net” format output, HistCite is only compatible with Web of Science output, and Gephi necessitates the use of a third-party programme for file preparation. As a result, we chose to utilize VOS viewer since it does not need any preparation and has been extensively used in network research for many years. For our analysis, we used of the lens.org dataset.

**Figure 13:**
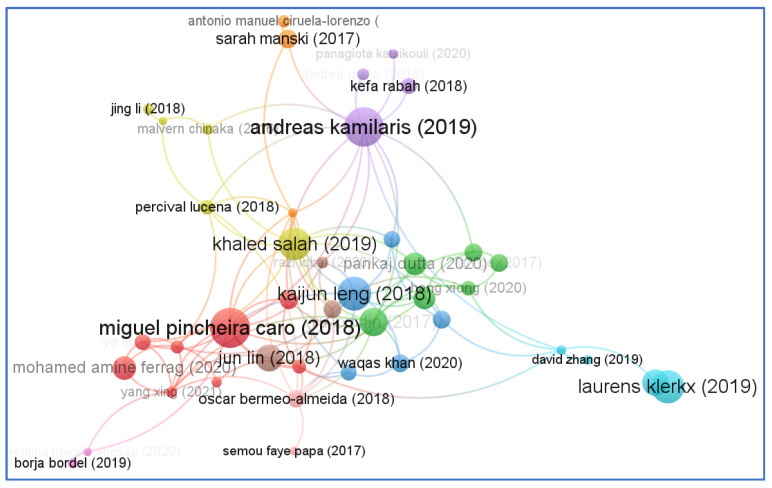
Citation network based on documents.

**Figure 14:**
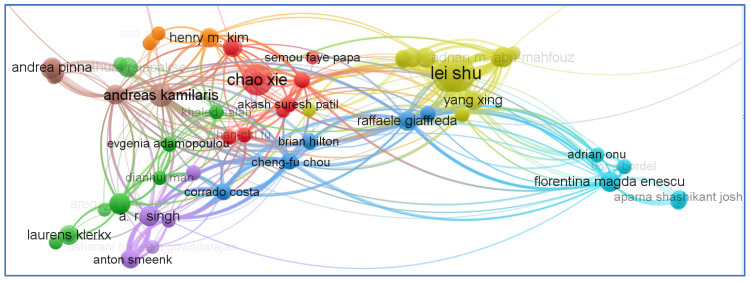
Citation’s network based on authors.

Direct citation analysis is a statistical study that indicates how many times an article has been mentioned in other papers. In addition to “documents”, “author”, and “source”, VOS viewer includes a variety of other units of analysis. In order to facilitate citation network analysis, we chose “documents” from the database as a unit of analysis. It is clear from Figure 13 that the documents of Andreas Kamilaris (2019) have the greatest impact in terms of citations. Secondly, we chose “author” as another unit of analysis to further support the co-authorship and “document” citation analyses. Figure 14 shows the analysis network based on documents. 

**Figure 15:**
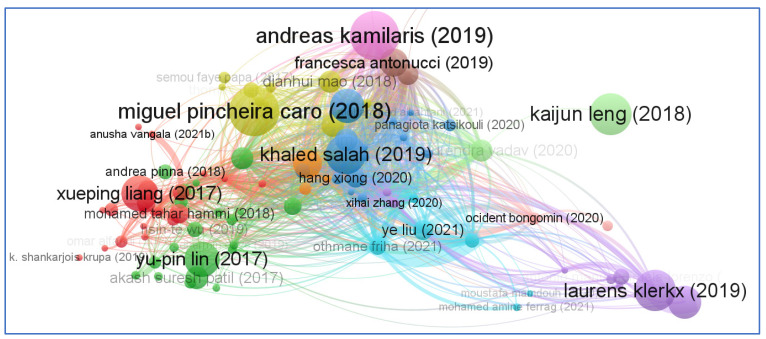
Bibliographic coupling network based on documents.

**Figure 16:**
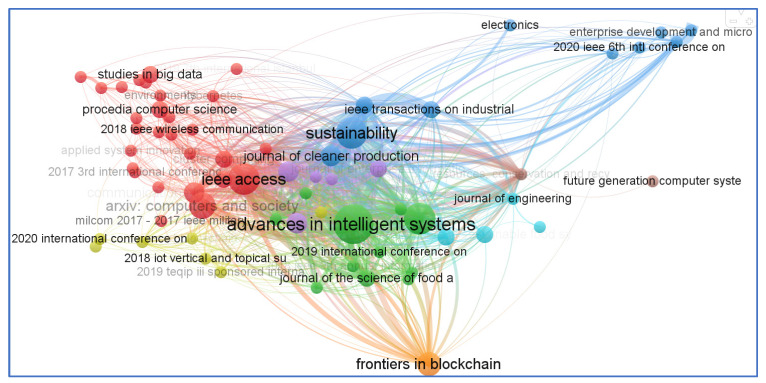
Bibliographic coupling network based on sources.

**Figure 17:**
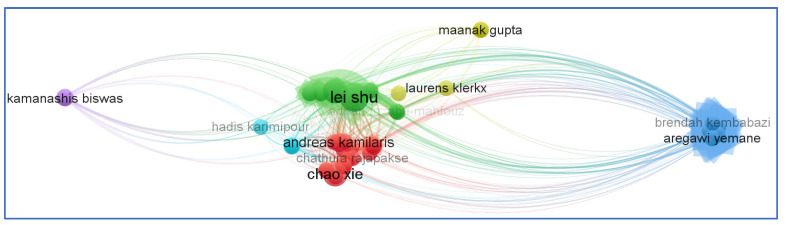
Bibliographic coupling network based on authors with a minimum of 12 citations.

One of the oldest citation analysis approaches for document similarity measurement is known as ‘Bibliographic Coupling’. M.M. Kessler of the Massachusetts Institute of Technology was the first to propose this notion in 1963. When two papers contain similar references, they are considered bibliographically connected. In other words, bibliographic coupling occurs when mutually cited references can be identified in their bibliographies. The coupling intensity is considered to be stronger when the two-referencing works have more citations in common than not. This connection also impacts the degree to which the two works are comparable in terms of subject matter. Bibliographic coupling is equally beneficial since it assists researchers in locating previous research that is relevant to their current research. VOS Viewer tool is widely used to analyze bibliographic coupling networks. It is capable of supporting three different kinds of bibliographic coupling networks, the first of which uses “documents” as the unit of analysis, the second uses “sources” as the unit of analysis, and the third uses “author” as the unit of analysis. Our analysis of the aforementioned bibliographic connection network is shown in Figure 15. This analysis was created with “documents” serving as the unit of analysis. Another bibliographic connection network analysis can be seen in Figure 16. This analysis was created with “sources” serving as the unit of analysis. Figure 17 shows the bibliographic coupling analysis with author as the analysis unit. During the analysis, we selected only those authors who have atleast twelve citations. 

### 4.2. Classification of Papers and Review

**Figure 18:**
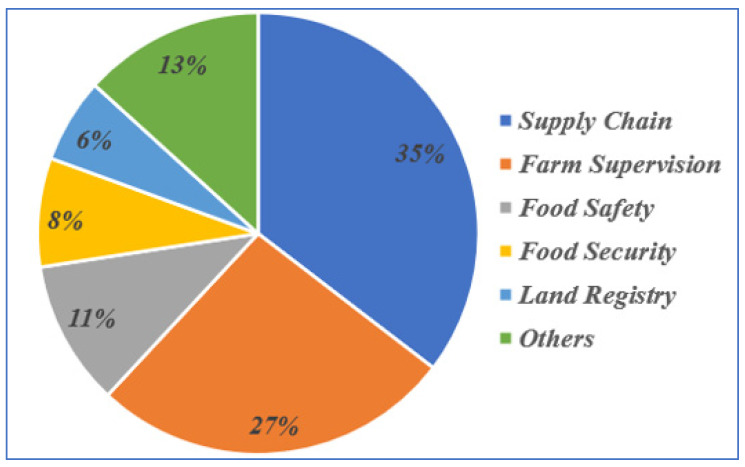
Percentage distribution of the selected papers.

As each article addresses one or more blockchain use cases in agriculture, we decided to categorize them by use case. Figure 18 shows that 35% of the 118 papers covered agricultural supply chain use cases. From Figure 18, it can also be seen that 27% of the papers concentrated on blockchain-based agricultural oversights, 11% covered food safety, and 8% discussed food security. Additionally, 6% of the selected papers discussed land registration using BCT. The blockchain-related use cases discussed in the literature are detailed in subsequent subsections.

#### 4.2.1. Farm Supervision

In today’s world, technology can help revolutionize and create new perspectives on farm management. Various state-of-the-art technologies can be used to create smart and intelligent farms. Sensor-based techniques can be used in combination with IoT devices to forecast weather conditions among other things (e.g., maturity sensors for crops). Storing agricultural data digitally using sensors, offers stakeholders and farmers convenient access to information for various purposes. By implementing blockchain technology, sensor networks can be managed more efficiently and effectively [9,10]. Additionally, BCT can be used to trace carbon dioxide levels, potentially helping to avoid the growth of fungus.

#### 4.2.2. Land Registry

Land registration is a process of defining a system in which the respective ownership, value, use, and all rights related to that property are explained in a proper way. Currently, the system used for land registry has lots of restrictions. It is impossible to completely authenticate and verify transactions between the land owner and a person or organization [11,12]. All of these problems can be solved by using BCT as it could avoid uncertainty and establish trust by helping to maintain the land registration data. As the blockchain is based on the concepts of a distributed ledger system and a decentralized environment, it becomes easy to store all of the land registry details, for example, timestamped data, transactions, previous ownership, etc. [13,14].

#### 4.2.3. Food Safety

Food safety is an important factor in managing the quality of food. Obviously, food is a vital resource and is crucial to human health. Therefore, we should concentrate on the environment used to manage and store food products. Additionally, food contamination can cause illnesses. Blockchain technology could become help in providing solutions to these issues. With the help of blockchain technology, improved food traceability could be achieved [15,16,17]. Blockchain provides information regarding the food distribution process, including information on the starting point and the path required to reach the seller and consumer, while in traditional food distribution methods, it can take more than six days to obtain information about the path that products have to follow.

#### 4.2.4. Supply Chains

A supply chain describes the network in which the movement of products from farm to consumer is managed. In the current system, the product keeps moving down the chain for a long time, and it is not easy to track the supply chain. However, with the implementation of blockchain technology, this problem can be solved, and consumers can trace the quality of the products [18,19]. This may help to improve the belief in agricultural products and reduce fraud regarding product nutrition quality [20].

#### 4.2.5. Food Security

Basically, food security can be defined as ensuring that every individual has social, physical, and convenient access to healthy food at any time. This includes maintaining the nutritional value of the food and monitoring pesticide levels in crops. Blockchain technology offers a solution to address these challenges [21,22]. By utilizing blockchain, all relevant product information can be shared with consumers [23,24,25]. By enhancing the supply chain, blockchain can contribute to the improvement of food security.

## 5. Discussion and Research Questions

1. What is the current state of research on the development of BCT in the agrifood industry?

Increased interest in BCT and an improved understanding of its potential applications the agrifood sector can be seen in the existing literature. Researchers and industry professionals have explored the use of BCT in a number of business-related areas, including supply chain management, traceability, quality control, and smart contracts. As previously stated in the present study, BCT has the ability to improve security, efficiency, and transparency in the agrifood industry by tackling problems regarding food fraud, supply chain opacity, and consumer trust issues.

2. What are the use cases for Blockchain in agriculture?

This study found a variety of applications for blockchain in agriculture. Some noteworthy use cases include the following:

1. Supply chain transparency and traceability: Blockchain technology may be utilised to increase transparency and traceability across the agricultural supply chain. Stakeholders may confirm the source, procedures of production, quality, and management of agricultural products by documenting all transactions and the movement of items on the blockchain. 

2. Certification and authentication: Using blockchain, it is possible to produce tamper-proof records of certifications and authentications pertaining to sustainable development, fair trade, organic agricultural practises, and other quality requirements. 

3. Smart contracts for fair trade: Automated, secure, and transparent agreements between farmers and buyers can be made possible by blockchain-based smart contracts. These agreements may guarantee honest pricing, fast payments, and effective trade transactions, therefore lowering reliance on middlemen and enhancing farmers’ quality of life.

4. Land and crop ownership: A decentralised, immutable ledger for tracking land ownership and crop management rights may be provided via blockchain. This may assist in preventing land conflicts, enabling open business dealings, and improving farmers’ access to loans and insurance.

5. Agricultural insurance: By automating verification claims and safely preserving policy information, blockchain technology can help expedite the agricultural insurance process. Blockchain’s decentralised structure guarantees transparency, lowers fraud, and expedites the claim settlement process.

6. Decentralised marketplaces: By cutting out middlemen and reducing expenses, blockchain-based marketplaces may link farmers with customers directly. These markets promote fair trade and give farmers more control by enabling transparent pricing, direct feedback, and safe transactions.

7. Data management and sharing: Agricultural data, such as weather patterns, soil quality, and crop yields, may be safely stored and shared using blockchain technology. This facilitates data-based decision making, improves research cooperation, and encourages agricultural innovation.

3. What are the primary research themes and topics related to the application of BCT in the agrifood industry?

The primary research themes and areas of study surrounding the use of BCT in the agricultural and food sectors are as follows: A key theme is supply chain traceability. Much of the research we found concentrated on using BCT to enhance the traceability of agricultural goods, allowing for the precise monitoring of a product’s origin, manufacturing methods, and distribution. This subject seeks to increase accountability and decrease fraud in supply chains. Another key theme is quality control and certification. BCT has been proposed as a way to enhance the agrifood sector’s quality control procedures. Researchers want to use BCT to create an unchangeable record of certifications, audits, and quality control data, ensuring the safety and integrity of food items and fostering trust amongst sellers and customers.

4. What are the challenges and limitations of the blockchain-based applications?

The following are some problems and limitations with blockchain-based applications in agriculture: (a) Scalability: BCT systems often struggle with scaling, particularly when dealing with the many transactions and data points that are present in the agricultural supply chain. (b) Interoperability: It may be challenging to integrate blockchain technology with current systems and databases, necessitating the use of interoperability standards and protocols.

5. What are the open research issues and the areas for future research?

Open research questions and future research directions regarding the incorporation of BCT into the agrifood sector include the following:

(a) Governance models: Investigating governance models and frameworks for BCT implementation in the agricultural sector to address concerns related to decision-making processes, consensus procedures, and regulatory compliance.

(b) Sustainability and environmental impact: Analysing the possible environmental implications of applying BCT to agriculture and looking for strategies to lessen any unfavourable outcomes.

(c) User adoption and acceptance: Recognising the elements that may affect how stakeholders in the agricultural and food industries accept and embrace blockchain-based solutions.

(d) Integration with emerging technologies: Investigating how BCT may be integrated with other cutting-edge technologies, such as the Internet of Things (IoT) and Artificial Intelligence (AI), to improve the efficacy and efficiency of agricultural systems.

(e) Economic and social implications: Analysing the economic and social ramifications of BCT implementation in the agricultural sector, including its impact on small-scale farmers, market dynamics, and overall industry sustainability.

## 6. Conclusions

This study presented an in-depth bibliometric study on the development and use of BCT in the agrifood sector. This study discussed the present state of research, key research issues, use cases, obstacles, and future research objectives in this subject by analysing a broad variety of academic publications published between 2014 and 2022. The results of this study demonstrate how researchers are becoming more interested in examining the possibility of incorporating BCT into the agrifood supply chain. BCT has established itself as a highly unique idea, drawing the interest of academics and professionals from a variety of fields. The use of BCT in agriculture presents several prospects for enhancing supply chain efficiency, traceability, and transparency. There are a number of important applications for BCT in the agrifood industry, including those associated with supply chain transparency and traceability, certification and authentication, smart contracts for fair trade, the ownership of land and crops, agricultural insurance, decentralised marketplaces, and data management and sharing. These potential use cases show how BCT has the capacity to solve major problems in the industry and transform the market. However, the use of BCT in agriculture also has drawbacks and difficulties. Interoperability and scalability are crucial topics that require further study. To maximise the advantages of BCT and ensure its successful integration into agricultural systems, it is essential to overcome these difficulties. Future research should examine governance frameworks for BCT deployment, evaluate BCT’s sustainability and environmental effect in agriculture, comprehend user uptake and acceptability, and combine BCT with cutting-edge technologies like IoT and AI. These avenues for study will help us to better comprehend BCT’s potential in the agricultural and food sectors. Overall, this study presents insightful information on the current status of BCT usage in agriculture and offers recommendations for policymakers, industry stakeholders, and academics who are interested in using BCT to solve issues and seize opportunities in the agrifood sector. We believe a more open, effective, and sustainable agricultural landscape can be created by adopting BCT and furthering research in this area.

## Figures and Tables

**Table 1:** Top institutions by country.

Institution Country/Region	Institution Name	Avg. Citations
China	Chinese Academy of Sciences	77
United States	Old Dominion University	59
Australia	Commonwealth Scientific and Industrial Research Organization	54
United Kingdom	De Montfort University	52
United Kingdom	Nanjing Agricultural University	41
China	Nanjing Agricultural University	33
United States	Agricultural & Applied Economics Association	24
India	National Institute of Industrial Engineering	23
India	National Institute of Technology, Raipur	23
China	City University of Hong Kong	19
India	International Institute of Information Technology, Hyderabad	16
Australia	Australian Catholic University	11
Australia	Griffith University	11
Australia	Open University	11
Australia	Shahjalal University of Science and Technology	11

**Table 2:** Top 10 funding agencies.

Average Reference Count	Publication Year	Funding
128	2021	5GEAR Menot CWC-NS
128	2021	6Genesis Flagship
123	2021	China Postdoctoral Science Foundation
121	2021	Academy of Finland
87	2021	ARC
69	2021	National Natural Science Foundation of China
27	2019	AGROENER
27	2019	AgroFiliere (AgriDigit program)
27	2020	AIC-IIITKottayam

**Table 3:** Top fields of study in terms of document count.

Field of Study	Document Count
Blockchain	194
Business	181
Agriculture	164
Computer science	161
Supply chain	96
Traceability	73
Environmental economics	49
Industrial organization	38
Internet of Things	37
Smart contract	37
Supply chain management	34
Database transaction	30
Big data	24
Cloud computing	22
Emerging technologies	22

## Data Availability

No external datasets were used, and all the references are duly cited.
